# The Circular RNA hsa_circ_0001445 Regulates the Proliferation and Migration of Hepatocellular Carcinoma and May Serve as a Diagnostic Biomarker

**DOI:** 10.1155/2018/3073467

**Published:** 2018-01-23

**Authors:** Xianwei Zhang, Hu Zhou, Wei Jing, Ping Luo, Shili Qiu, Xuefang Liu, Man Zhu, Chunzi Liang, Mingxia Yu, Jiancheng Tu

**Affiliations:** ^1^Department & Program of Clinical Laboratory Medicine, Center for Gene Diagnosis, Zhongnan Hospital of Wuhan University, Wuhan 430071, China; ^2^Department of Blood Transfusion, Tongji Hospital, Tongji Medical College, Huazhong University of Science and Technology, Wuhan 430030, China

## Abstract

Circular RNAs (circRNA), a class of noncoding RNAs, have been found to be involved in various diseases. Here, the expression levels of the circRNA hsa_circ_0001445 in 73 pairs of hepatocellular carcinoma (HCC) and adjacent nontumor tissues were investigated by quantitative real-time polymerase chain reaction (qRT-PCR). Our data demonstrate that the hsa_circ_0001445 levels were significantly decreased in HCC tissues (*P* < 0.001) and markedly associated with the number of tumor foci (*P* = 0.014). Furthermore, *in vitro* approaches showed that overexpression of hsa_circ_0001445 promoted apoptosis and inhibited proliferation, migration, and invasion of HCC-derived cells, suggesting that hsa_circ_0001445 might be involved in the development of HCC. In addition, we found that the plasma hsa_circ_0001445 transcription levels in HCC patients were lower than those in cirrhosis (*P* < 0.001) and hepatitis B (*P* < 0.001) patients as well as in healthy controls (*P* < 0.001). In fact, receiver operating characteristic curve analysis indicated that plasma hsa_circ_0001445 could be a fairly accurate marker to distinguish HCC cases from healthy controls as well as patients with cirrhosis or hepatitis B.

## 1. Introduction

Hepatocellular carcinoma (HCC) is the third leading cause of cancer-related mortality worldwide [1]. HCC usually develops in cirrhotic livers, which mainly result from chronic hepatitis B virus (HBV) infections in Asia [2]. For decades, the alpha-fetoprotein (AFP) has been one of the most commonly used biomarkers for HCC diagnosis. However, owing to their poor sensitivity and specificity, the current AFP-based diagnostic approaches are far from being satisfactory [3]. Therefore, there is an urgent need to develop better biomarkers for HCC diagnosis.

Circular RNAs (circRNAs) are a class of endogenous noncoding RNAs that result from a noncanonical form of alternative splicing [4]. Unlike typical RNAs that are linear, the 5′ and 3′ terminals of circRNAs are joined to form closed loops [5]. Although circRNAs were first reported in the 1970s [6], they were misconstrued as products of transcriptional noise in eukaryotes [7], owing to which little further research was reported for the next decades. Owing to recent advances in high-throughput sequencing and bioinformatics, the functions of circRNA have been reexamined. Studies have revealed that circRNAs can serve as microRNA (miRNA) sponges to sequester miRNAs from their bound target genes [8–11]. As a result, circRNAs can inhibit miRNA functions and could play important roles in various cellular activities and disease processes [12–14]. In addition, studies have shown that circRNAs might also act as biomarkers for many types of cancers [15–17], including HCC. For instance, the circRNA MTO1 was significantly downregulated in HCC tissues, and the low expression of circRNA MTO1 was considered as a poor prognosis marker for HCC patients [18].

Recently, Conn et al. [19] have shown that levels of hsa_circ_0001445 (also named circSMARCA5) were significantly increased with a minimal change in the levels of linear mRNA during epithelial-to-mesenchymal transition (EMT). As a well-known crucial step of HCC, EMT accelerates tumor progression by enhancing metastasis [20–23]. Based on the above information, we attempted to investigate the relationship between hsa_circ_0001445 levels and HCC. First, we measured the expression levels of hsa_circ_0001445 in HCC and adjacent nontumor tissues. Then, *in vitro* experiments were performed to explore the biological function of hsa_circ_0001445. Finally, we analyzed the plasma levels of hsa_circ_0001445 in HCC, cirrhosis, hepatitis B patients, and healthy controls to determine its diagnostic value for the detection of HCC.

## 2. Materials and Methods

### 2.1. Patient Data and Specimen Collection

A total of 73 pairs of HCC and adjacent nontumor tissues were obtained from HCC patients (67 males and 6 females, mean age 54 ± 10) who underwent surgery without preoperative chemotherapy or radiotherapy in Zhongnan Hospital of Wuhan University from 2011 to 2015. All patients were selected based on their pathology reports. Tumor staging (stages I, II, III, and IV) was defined according to the seventh edition of the AJCC Cancer Staging Manual. The number of tumor foci was determined by computed tomography and pathology reports. Tumor specimens and paired adjacent nontumor tissues were stored at −80°C in RNAlater® RNA Stabilization Solution (Invitrogen, USA). Blood samples from 104 HCC patients (87 males and 17 females, mean age 59 ± 11), 57 cirrhosis patients (45 males and 12 females, mean age 56 ± 9), 44 hepatitis B patients (33 males and 11 females, mean age 51 ± 15), and 52 healthy subjects (39 males and 13 females, mean age 54 ± 13) were obtained from Zhongnan Hospital of Wuhan University, between 2016 and 2017. All the healthy subjects chosen for the study were free of hepatitis, hepatic diseases, or abnormal liver biochemical outcomes. The blood samples were collected in EDTA-anticoagulant tubes and centrifuged at 2000*g* for 5 min at 4°C. The supernatants obtained were transferred to microcentrifuge tubes and centrifuged at 12,000*g* for 5 min at 4°C for a complete removal of the cell debris. The obtained plasma samples were stored at −80°C until used.

### 2.2. RNA Extraction and Reverse Transcription

Total RNA content of tissues and plasma were extracted using the Trizol reagent (Invitrogen, USA) and blood total RNA isolation kit (Bioteke, China), respectively, according to the manufacturers' instructions. The concentration and purity of the obtained RNA samples were quantified using the NanoDrop ND2000 (Thermo, USA). The RNA samples were reverse-transcribed to cDNA using the PrimeScript™ RT reagent kit with gDNA Eraser (Takara, Japan) according to the manufacturer's instructions.

### 2.3. Quantitative Real-Time Polymerase Chain Reaction (qRT-PCR) Techniques

The expression levels of hsa_circ_0001445 were detected via qRT-PCR with the Bio-Rad CFX96 (Bio-Rad, USA) according to the manufacturer's instructions. The reactions were started with an initial denaturation at 95°C for 5 min, followed by denaturation at 95°C for 30 s, annealing at 63.3°C for 30 s, and extension at 72°C for 30 s. The denaturation, annealing, and extension steps were repeated for 40 cycles. The glyceraldehyde 3-phosphate dehydrogenase (GAPDH) gene was used as an internal control. The primers used for the PCR reactions were hsa_circ_0001445 (forward: 5′-CAAGATGGGCGAAAGTTCACT-3′ and reverse: 5′-TGTGTTGCTCCATGTCTAATCATT-3′) and GAPDH (forward: 5′-AGAAGGCTGGGGCTCATTTG-3′ and reverse: 5′-GCAGGAGGCATTGCTGATGAT-3′). All experiments were carried out in duplicate for each data point.

### 2.4. Plasmid Construction

We used the PcDNA3.1(+) circRNA (Addgene plasmid number 60648), a mini vector that is a circRNA-forming plasmid, a kind gift from Jeremy Wilusz [24] to subclone the hsa_circ_0001445 sequence for the transfection studies. The hsa_circ_0001445 cDNA was amplified by PCR with the following primers: forward, 5′-TTAATTAAGGAGGCTTGTGGATCAGAAT-3′ and reverse, 5′-TCCCCGCGGCTTTTGTTTTTCTCTATAGT-3′. The obtained PCR fragment was subcloned into the PacI and SacII sites of the PcDNA3.1 (+) circRNA mini vector. Afterwards, the reconstructed plasmid was validated by DNA sequencing. Finally, more copies of the reconstructed plasmids were produced and purified using the Mini Plasmid Preparation Kit (Axygen, China) according to the manufacturer's instructions.

### 2.5. Cell Culture and Plasmid Transfection

The HCC cell lines HepG2, HCCLM9, Hep3B, HCCLM3, and MHCC97L as well as the immortalized human hepatic cell line L02 were obtained from the Cell Bank of Type Culture Collection of Chinese Academy of Sciences (Shanghai, China). Cells were cultured in DMEM (Gibco, USA) with 10% fetal bovine serum (Gibco, USA) in a humidified incubator at 37°C with 5% CO_2_. A six-well plate was seeded with 5 × 10^5^ cells and incubated for 24 h, the cells were then transfected with pcDNA3.1(+)-circRNA-hsa_circ_0001445 or pcDNA3.1(+) circRNA mini vector using Lipofectamine™ 2000 (Invitrogen, USA) according to the manufacturer's instructions.

### 2.6. Cell Proliferation Assay

Cell proliferation assays were conducted using the Cell Counting Kit-8 (CCK-8) (Dojindo, Japan) according to the manufacturer's instructions. In brief, transfected cells were seeded into 96-well plates (2000 cells/well) and cultured for 0 h, 24 h, 48 h, and 96 h. Then, 10 *μ*l of CCK8 solution was added to each well, and the plates were incubated at 37°C for additional 2 h. Finally, the solution was measured using a 450 nm spectrophotometer (EnSpire, PerkinElmer, USA).

### 2.7. Flow Cytometric Analysis

Transfected HepG2 cells were harvested after transfection for 24 h and stained using an Annexin V-FITC/PI apoptosis detection kit (Beyotime, China) according to the manufacturer's instructions. The cells were then analyzed with a Cytomics™FC500 flow cytometer (Beckman Coulter, USA).

### 2.8. Cell Migration and Invasion Assays

Transfected HepG2 cells were harvested after transfection for 24 h and seeded into the upper chambers (20,000 cells/chamber) of transwell assay plates (Corning, USA) with 200 *μ*l of serum-free DMEM to quantify cell migration. Similarly, transfected HepG2 cells were seeded into the upper chambers (40,000 cells/chamber) of transwell plates with the Matrigel-coated membrane (BD, USA) in 200 *μ*l serum-free DMEM to quantify cell invasion. The lower chambers were filled with DMEM containing 10% FBS. After an incubation period of 24 h, the medium was removed, and cells were fixed with methanol for 20 min. The cells were then stained with crystal violet for 20 min. Air-dried and photographed with a digital microscope. The number of cells was calculated from five random fields for each chamber.

### 2.9. Statistical Analyses

All statistical analyses were carried out with SPSS version 21.0 (SPSS Inc., USA) and GraphPad Prism 5.0 (GraphPad software, USA). Normally distributed data are presented as mean ± standard error of mean (M ± S.E.M.). Results were considered statistically significant for *P* < 0.05. The normality of distribution for each data set was tested by the Shapiro-Wilk test. Normally distributed data sets were analyzed by Student's *t*-tests, while nonnormally distributed data were analyzed by Kruskal-Wallis variance analyses. Correlations were analyzed by the Spearman correlation method. The combined diagnosis of hsa_circ_0001445 and AFP was analyzed using binary logistic regression. Finally, receiver operating characteristic (ROC) curves were generated to assess the diagnostic value of hsa_circ_0001445.

## 3. Results

### 3.1. Expression of hsa_circ_0001445 Was Significantly Lower in HCC Tissues and Associated with the Number of Tumor Foci

**Figure 1:**
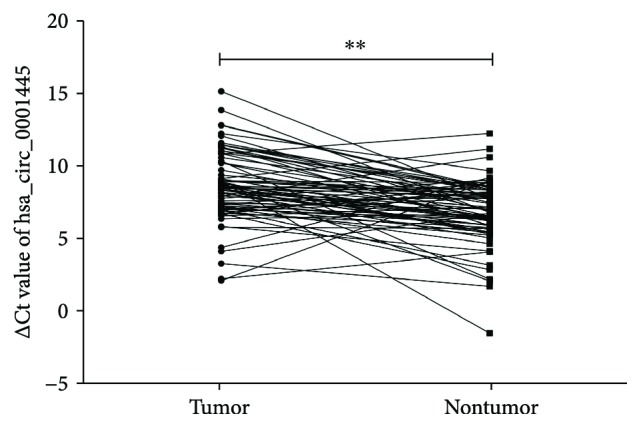
hsa_circ_0001445 was downregulated in HCC tissues compared to that in adjacent nontumor tissues. The ΔCt value was determined by subtracting the Ct value of GAPDH from the Ct value of the circRNA. Larger ΔCt values indicate lower expression. The data were analyzed using the paired Student's *t*-test. ^∗∗^ *P* < 0.01.

The expression of hsa_circ_0001445 was measured in 73 pairs of HCC and adjacent nontumor tissues by qRT-PCR (Figure 1). The results showed that the expression of hsa_circ_0001445 was significantly lower in HCC tissues compared to that in the adjacent nontumor tissues (*P* < 0.001). The analysis of the relationship between hsa_circ_0001445 expression and clinical characteristics of HCC was performed (Table 1). The results indicated that the expression of hsa_circ_0001445 was associated with the number of tumor foci in HCC patients (*P* = 0.014), while no statistically significant relationship was found between hsa_circ_0001445 expression and gender, age, smoking, alcoholism, tumor size, TNM stages, differentiation, AFP, or other biochemical indices.

### 3.2. Overexpression of hsa_circ_0001445 Promoted Apoptosis and Inhibited Proliferation, Migration, and Invasion of HCC In Vitro

**Figure 2:**
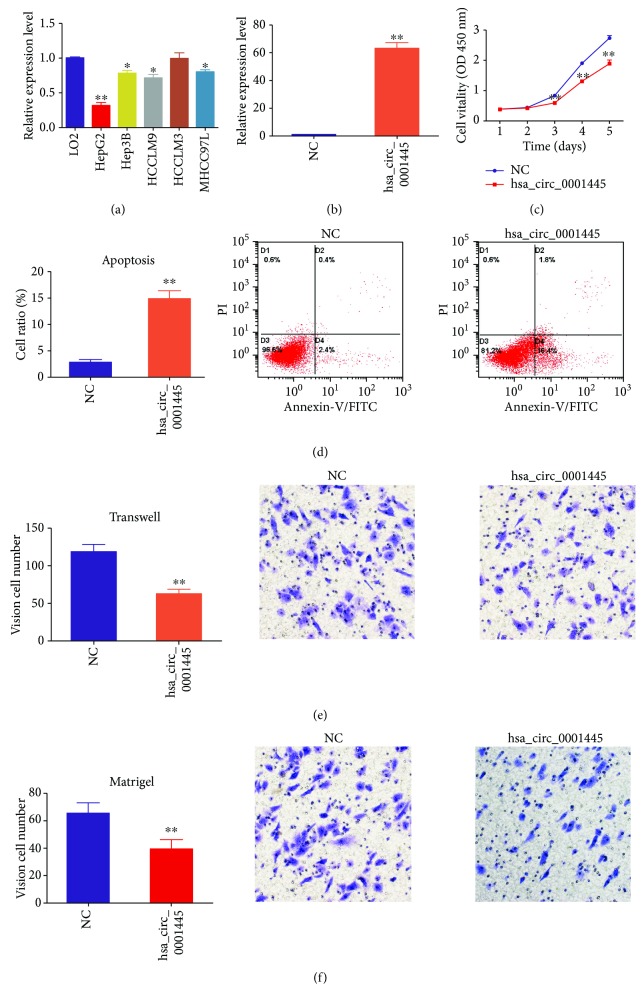
Overexpression of hsa_circ_0001445 promoted apoptosis and inhibited proliferation, migration, and invasion of HCC cells *in vitro*. (a) hsa_circ_0001445 expression levels were downregulated in the HCC-derived cell lines HepG2, Hep3B, HCCLM9, and MHCC97L. (b) The expression of hsa_circ_0001445 was significantly increased in HepG2 cells after transfection with pcDNA3.1(+)-circRNA-hsa_circ_0001445 for 24 h. (c) As indicated by the CCK8 assay, overexpression of hsa_circ_0001445 in HepG2 cells inhibited their proliferation. (d) Overexpression of hsa_circ_0001445 promoted apoptosis in HepG2 cells. (e) Overexpression of hsa_circ_0001445 decreased the migration capacity of HepG2 cells. (f) Overexpression of hsa_circ_0001445 impaired the invasion ability of HepG2 cells. ^∗^ *P* < 0.05, ^∗∗^ *P* < 0.01. Error bar indicates S.E.M. NC: negative control.

Next, we analyzed hsa_circ_0001445 expression in the HCC-derived cell lines and the hepatic cell line L02. The native expression of hsa_circ_0001445 in the HCC-derived cell lines HepG2, HCCLM9, Hep3B, and MHCC97L, but not HCCLM3, was significantly lower, compared to that in the hepatic cell line L02 (Figure 2(a)). To investigate the biological function of hsa_circ_0001445, we overexpressed hsa_circ_0001445 in HepG2 cells by transfection with pcDNA3.1(+)-circRNA-hsa_circ_0001445 (Figure 2(b)). In addition, SMARCA5, the host gene of hsa_circ_0001445, was detected in the hsa_circ_0001445 overexpressed cells by qRT-PCR. The results showed no significant difference in SMARCA5 mRNA expression between the hsa_circ_0001445 overexpressed cells and controls (Supplementary Figure 1).

The CCK8 assay showed that overexpression of hsa_circ_0001445 in HepG2 cells significantly inhibited their proliferation (Figure 2(c)). Subsequent flow cytometry results suggested that overexpression of hsa_circ_0001445 promoted apoptosis in these cells (Figure 2(d)). Data obtained from the transwell assay further indicated that overexpression of hsa_circ_0001445 impaired the migration capacity of HepG2 cells (Figure 2(e)). Consistently, the invasion ability of these cells was also diminished (Figure 2(f)). Together, our data suggest that hsa_circ_0001445 might serve as a tumor suppressor in HCC.

### 3.3. Plasma hsa_circ_0001445 Levels Were Lower in HCC Patients and Associated with Serum AFP Levels

**Figure 3:**
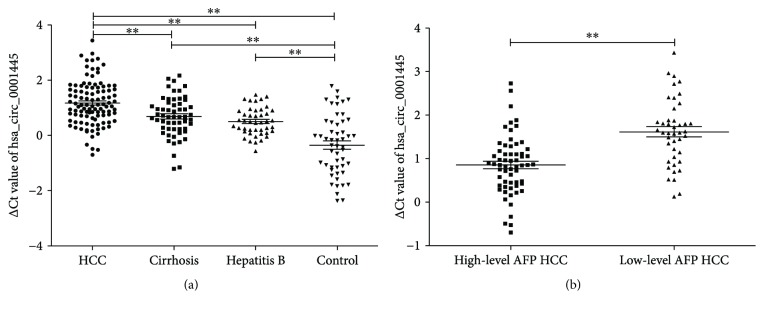
hsa_circ_0001445 expression levels in the plasmas of the different groups. (a) hsa_circ_0001445 expression levels in HCC patients were lower than those in cirrhosis and hepatitis B patients and healthy controls. (b) Plasma hsa_circ_0001445 was significantly lower in low-level serum AFP (<20 *μ*g/l) HCC patients than in high-level serum AFP (≥20 *μ*g/l) HCC patients. The ΔCt value was determined by subtracting the Ct value of GAPDH from the Ct value of the circRNA. A larger ΔCt value indicates a lower expression. The data was analyzed using Student's *t*-test. The results were expressed as mean ± S.E.M. ^∗∗^ *P* < 0.01. AFP: alpha-fetoprotein; HCC: hepatocellular carcinoma.

To assess the diagnostic potential of hsa_circ_0001445 for the detection of HCC, plasma hsa_circ_0001445 levels of 104 HCC patients, 57 cirrhosis patients, 44 hepatitis B patients, and 52 healthy controls were analyzed by qRT-PCR (Figure 3). The results indicated that plasma hsa_circ_0001445 levels of HCC patients were significantly lower than those of healthy controls (*P* < 0.001), cirrhosis patients (*P* < 0.001), or hepatitis B patients (*P* < 0.001). The expression of plasma hsa_circ_0001445 in cirrhosis (*P* < 0.001) and hepatitis B patients (*P* < 0.001) was also lower than that in the healthy controls. No significant difference in plasma hsa_circ_0001445 levels was found between cirrhosis and hepatitis B patients. Correlation analysis results (Table 2) showed that plasma hsa_circ_0001445 levels in HCC patients were associated with serum AFP levels (*P* = 0.009) (Figure 3(b)), while no statistically significant relationship was detected between hsa_circ_0001445 and gender, age, alanine aminotransferase (ALT), aspartate aminotransferase (AST) levels, or other biochemical indices.

### 3.4. Diagnostic Value of Plasma hsa_circ_0001445 Levels Alone and in Combination with Serum AFP Levels for the Detection of HCC

**Figure 4:**
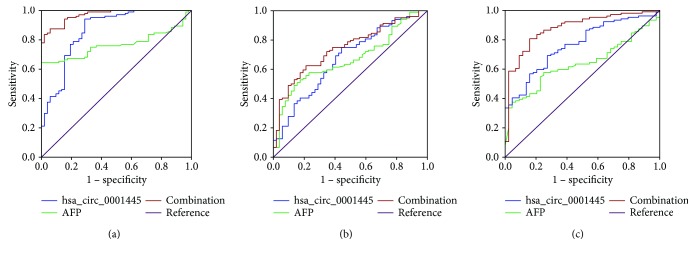
Diagnostic value of plasma hsa_circ_0001445 levels, serum AFP alone, and the combination of these two markers for the detection of HCC. (a) ROC curves of plasma hsa_circ_0001445, serum AFP alone, and the combination of these two markers to distinguish HCC patients from healthy controls. (b) ROC curves of plasma hsa_circ_0001445, serum AFP alone, and the combination of these two markers to distinguish HCC patients from cirrhosis patients. (c) ROC curves of plasma hsa_circ_0001445, serum AFP alone, and the combination of these two markers to distinguish HCC patients from hepatitis B patients. AFP: alpha-fetoprotein.

ROC curves were constructed to assess the diagnostic value of plasma hsa_circ_0001445 levels for HCC detection. The results indicated that the levels of plasma hsa_circ_0001445 can serve well as an indicator to determine HCC. To distinguish HCC patients from healthy controls (AUC = 0.862, 95% CI = 0.710–0.845), the specificity and sensitivity of using plasma hsa_circ_0001445 levels as diagnostic index were 94.2% and 71.2%, respectively (Figure 4(a)). Furthermore, plasma hsa_circ_0001445 levels could also be used to distinguish HCC patients from cirrhosis (AUC = 0.672, 95% CI = 0.586–0.758) (Figure 4(b)) and hepatitis B patients (AUC = 0.764, 95% CI = 0.686–0.842) (Figure 4(c)). Since AFP is the most commonly used diagnostic biomarker of HCC, and we had found that the expression of plasma hsa_circ_0001445 was associated with serum AFP of HCC, the combined diagnostic value of these two indicators was analyzed by stepwise logistic regression models (Supplementary Table 1). The efficiency of the combined diagnosis in distinguishing HCC cases from healthy controls (AUC = 0.970, 95% CI = 0.949–0.991), from cases of cirrhosis (AUC = 0.743, 95% CI = 0.664–0.821), or from cases of hepatitis B (AUC = 0.877, 95% CI = 0.817–0.938) was higher when compared to that using plasma hsa_circ_0001445 levels or serum AFP levels alone (Table 3). Collectively, our data supported that hsa_circ_0001445 might potentially serve as a novel diagnostic biomarker for HCC detection.

## 4. Discussion

Previous studies have revealed that circRNAs may be involved in the development of variety of cancers [25]. In this study, we reported that the expression levels of the circRNA hsa_circ_0001445 were significantly lower in HCC tissues compared to those in adjacent nontumor tissues and were associated to the number of tumor foci. Conn et al. revealed that the hsa_circ_0001445 expression is promoted by the RNA-binding protein Quaking (QKI), which binds to RNA sequences upstream and downstream of the circRNA-forming exons to facilitate the formation of the circRNAs [19]. Suppression of QKI transcription was previously found in colon, gastric, and prostate cancers [26–28], suggesting that hsa_circ_0001445 expression might be regulated similarly via QKI in HCC. However, a study by Ding et al. [29] reported that no significant change in QKI levels is found in HCC tissues, indicating that the molecular mechanism of hsa_circ_0001445 downregulation in HCC tissues warrants further investigation.

Consistent with our findings in paired HCC and adjacent nontumor tissue samples, we found that HCC-derived cell lines HepG2, Hep3B, HCCLM9, and MHCC97L exhibited lower expression levels of hsa_circ_0001445 compared to the hepatic cell line L02. Furthermore, our *in vitro* experiments showed that overexpressed hsa_circ_0001445 promoted the apoptosis and inhibited the proliferation in HepG2 cells. In addition, we found that overexpressed hsa_circ_0001445 inhibited the migration and invasion of HCC cells, indicating that hsa_circ_0001445 might inhibit the metastasis of HCC. Multifocal HCC is known to mainly resulted from intrahepatic metastasis [30, 31]. These findings coupled with our discovery of the negative relationship between tissue hsa_circ_0001445 expression and number of tumor foci suggest that lowered expression of tissue hsa_circ_0001445 might enhance intrahepatic metastasis and thus result in multiple tumor foci in HCC patients. Increasing evidence demonstrated that circRNAs have diverse biological functions and play important roles in cellular activities, such as functioning as miRNA sponges [8], regulating the RNA-binding proteins [32], and modulating the expression of parental genes [33]. However, the molecular mechanism of hsa_circ_0001445 in regulating HCC needs to be further investigated in the future.

Current studies indicated that noncoding RNAs (ncRNAs), including miRNAs [34], long noncoding RNAs [35], and circRNAs [18] could be used as biomarkers for HCC. In this study, we investigated the diagnostic value of plasma hsa_circ_0001445 levels for the detection of HCC for the first time. Our results demonstrated that plasma hsa_circ_0001445 levels were significantly lower in HCC patients than in healthy controls and in cirrhosis or hepatitis B patients. The ROC curves obtained in our analyses supported that the levels of plasma hsa_circ_0001445 show a good diagnostic value to distinguish HCC patients from healthy controls as well as patients with cirrhosis or hepatitis B. We also found that the expression of plasma hsa_circ_0001445 was associated with serum AFP levels in HCC patients. Although AFP is one of the most commonly used diagnostic biomarkers for HCC detection, the poor sensitivity and specificity of this biomarker have limited its clinical application. Surprisingly, by combining plasma levels of hsa_circ_0001445 and serum levels of AFP, the diagnostic efficacy for the HCC detection was remarkably improved, suggesting that hsa_circ_0001445 could serve as a complementary diagnostic biomarker to diagnose HCC instead of using AFP levels alone. However, a major limitation of our study is that it has been carried out on a small number of subjects. Therefore, our findings need to be validated by a large number of case trials.

## 5. Conclusion

Hsa_circ_0001445 levels were lower in HCC tissues than in adjacent nontumor tissues. Furthermore, *in vitro* studies indicated that hsa_circ_0001445 promoted apoptosis and inhibited proliferation, migration, and invasion in these cells. In addition, plasma levels of hsa_circ_0001445 could be a good diagnostic marker for differentiating HCC patients from healthy controls as well as from patients with cirrhosis or hepatitis B. Furthermore, plasma hsa_circ_0001445 and serum AFP levels, when used in combination, served as a remarkably sensitive diagnostic method for the detection of HCC. Collectively, our data support that hsa_circ_0001445 levels regulate HCC development and could serve as a potential diagnostic biomarker for HCC.

## Figures and Tables

**Table 1:** Relationship between tissue hsa_circ_0001445 expression (ΔCt) and clinical characteristics of HCC patients.

Characteristics	Patient number	Mean ± SD	*P* value
Tissues^∗∗^			<0.001
HCC	73	8.50 ± 0.32	
Adjacent	73	6.77 ± 0.77	
Gender			0.441
Female	6	8.41 ± 0.34	
Male	67	9.34 ± 0.97	
Age			0.100
<54	35	9.09 ± 0.45	
≥54	38	8.01 ± 0.49	
Smoking			0.562
Positive	42	8.43 ± 0.43	
Negative	22	8.01 ± 0.56	
Alcoholism			0.802
Positive	26	7.99 ± 0.54	
Negative	32	8.48 ± 0.37	
Tumor size			0.734
<5 cm	15	8.77 ± 0.48	
≥5 cm	54	6.23 ± 0.41	
Tumor foci^∗^			0.014
=1	32	7.30 ± 0.49	
>1	9	9.63 ± 0.70	
TNM			0.185
I/II	21	9.02 ± 0.36	
III/IV	40	8.16 ± 0.53	
Differentiation			0.163
High/moderate	56	8.25 ± 0.40	
Low	15	9.39 ± 0.42	
ALT			0.179
Negative	42	8.87 ± 0.41	
Positive	31	7.99 ± 0.50	
AST			0.798
Negative	35	8.58 ± 0.43	
Positive	32	8.41 ± 0.48	
AFP			0.339
Negative	35	8.13 ± 0.52	
Positive	35	8.77 ± 0.41	

The data are presented as mean ± S.E.M. ^∗^ *P* < 0.05, ^∗∗^ *P* < 0.01. The ΔCt value was determined by subtracting the Ct value of GAPDH from the Ct value of the circRNA. A larger ΔCt value indicates a lower expression. HCC: hepatocellular carcinoma; TNM: tumor-node metastasis; ALT: alanine aminotransferase; AST: aspartate aminotransferase; AFP: alpha-fetoprotein.

**Table 2:** Relationship between plasma hsa_circ_0001445 expression (ΔCt) and clinical characteristics of HCC patients.

Characteristics	Patient number	Mean ± SD	*P* value
Plasma^∗∗^			<0.001
HCC	55	1.02 ± 0.17	
Healthy controls	44	−0.70 ± 0.23	
Gender			0.202
Male	87	1.13 ± 0.21	
Female	17	0.45 ± 0.50	
Age			0.197
<59	52	1.08 ± 0.15	
≥59	52	1.25 ± 0.17	
ALT			0.272
Negative	61	0.99 ± 0.13	
Positive	43	1.15 ± 0.20	
AST			0.959
Negative	23	1.03 ± 0.28	
Positive	32	1.01 ± 0.28	
GGT			0.414
Negative	35	0.96 ± 0.17	
Positive	69	1.17 ± 0.15	
GLU			0.486
<6.1	84	1.14 ± 0.13	
≥6.1	19	0.93 ± 0.27	
AFP^∗∗^			0.009
Negative	43	1.46 ± 0.15	
Positive	61	0.85 ± 0.16	
CEA			0.359
Negative	93	1.06 ± 0.12	
Positive	11	1.40 ± 0.34	
Ca199			0.113
Negative	83	1.01 ± 0.23	
Positive	21	1.46 ± 0.42	
Ca125			0.231
Negative	72	1.01 ± 0.13	
Positive	32	1.31 ± 0.27	
HBV			0.210
<500	53	1.25 ± 0.19	
≥500	51	0.96 ± 0.14	

The data are presented as mean ± S.E.M. ^∗∗^ *P* < 0.01. The ΔCt value was determined by subtracting the Ct value of GAPDH from the Ct value of the circRNA. A larger ΔCt value indicates a lower expression. HCC: hepatocellular carcinoma; ALT: alanine aminotransferase; AST: aspartate aminotransferase; GGT: *γ*-glutamyl transferase; GLU: glucose; AFP: alpha-fetoprotein; HBV: hepatitis B virus.

**Table 3:** Diagnostic value of plasma hsa_circ_0001445 levels, serum AFP alone, and the combination of these two markers for the detection of HCC.

Biomarker	Subgroup	AUC	95% CI	Sen	Spe	*P*
hsa_circ_0001445	HCC versus control	0.862	0.796–0.927	0.942	0.712	<0.001
AFP	HCC versus control	0.767	0.694–0.840	0.644	1.000	<0.001
Combination	HCC versus control	0.970	0.949–0.991	0.875	0.942	<0.001
hsa_circ_0001445	HCC versus cirrhosis	0.672	0.586–0.758	0.740	0.544	<0.001
AFP	HCC versus cirrhosis	0.669	0.583–0.754	0.529	0.827	<0.001
Combination	HCC versus cirrhosis	0.743	0.664–0.821	0.625	0.788	<0.001
hsa_circ_0001445	HCC versus hepatitis B	0.764	0.686–0.842	0.692	0.727	<0.001
AFP	HCC versus hepatitis B	0.637	0.549–0.725	0.375	0.955	0.008
Combination	HCC versus hepatitis B	0.877	0.817–0.938	0.808	0.818	<0.001

Sen: sensitivity; Spe: specificity; HCC: hepatocellular carcinoma; AFP: alpha-fetoprotein.

## Abbreviations

HCC: Hepatocellular carcinoma
circRNA: Circular RNA
qRT-PCR: Quantitative real-time polymerase chain reaction
EMT: Epithelial-to-mesenchymal transition
ALT: Alanine aminotransferase
AST: Aspartate aminotransferase
GGT: *γ*-Glutamyl transferase
GLU: Glucose
AFP: Alpha-fetoprotein
HBV: Hepatitis B virus
NC: Negative control
ROC: Receiver operating characteristic
QKI: Quaking.

## Supplementary Materials

Supplementary 1 Figure 1: no significant difference of SMARCA5 mRNA expression was found in hsa_circ_0001445 overexpressed HCC cells compared with the negative control cells.

Supplementary 2 Table 1: the logistic models of combined diagnosis of plasma hsa_circ_0001445 and serum AFP for HCC detection.
