# Supplementary material for: The Circular RNA hsa_circ_0001445 Regulates the Proliferation and Migration of Hepatocellular Carcinoma and May Serve as a Diagnostic Biomarker
Source: Dis Markers. 2018 Jan 23;2018:3073467. doi: 10.1155/2018/3073467 (PMC5896272; doi:10.1155/2018/3073467)
Supplement: Supplementary 1 — Figure 1: no significant difference of SMARCA5 mRNA expression was found in hsa_circ_0001445 overexpressed HCC cells compared with the negative control cells. [file 3073467.f1.pdf]

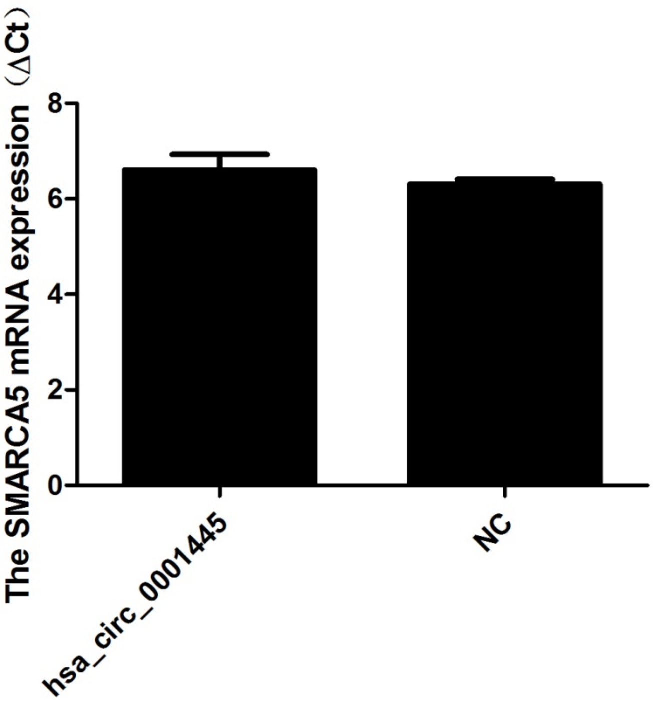

Supplementary Figure 1: No significant difference of SMARCA5 mRNA expression was found in hsa_circ_0001445 over-expressed HCC cells compared with the negative control cells.
