# Supplementary material for: The Circular RNA hsa_circ_0001445 Regulates the Proliferation and Migration of Hepatocellular Carcinoma and May Serve as a Diagnostic Biomarker
Source: Dis Markers. 2018 Jan 23;2018:3073467. doi: 10.1155/2018/3073467 (PMC5896272; doi:10.1155/2018/3073467)
Supplement: Supplementary 2 — Table 1: the logistic models of combined diagnosis of plasma hsa_circ_0001445 and serum AFP for HCC detection. [file 3073467.f2.docx]

**Supplementary table 1:** The logistic models of combined diagnosis of plasma hsa_circ_0001445 and serum AFP for HCC detection.

| Subgrup | Logistic model |
| --- | --- |
| HCC vs Controls | logit(p)=-2.805+0.246×(AFP)+2.348×(hsa_circ_0001445) |
| HCC vs Cirrhosis | logit(p)=-1.133+0.003×(AFP)+1.316×(hsa_circ_0001445) |
| HCC vs Hepatitis B | logit(p)=-1.479+0.004×(AFP)+2.070×(hsa_circ_0001445) |

Abbreviations: HCC, hepatocellular carcinoma; AFP, alpha-fetoprotein.
